# Rationale, Design, and Implications of Bifurcation Coronary Stenting: Insight from the Indian Bifurcation Stenting (IBIS) Registry

**DOI:** 10.7759/cureus.18027

**Published:** 2021-09-16

**Authors:** Jabir A, Amit Malviya, Joby Thomas, Vijaykumar S, Ashishkumar Mandalay, Jo Joseph, Rony Mathew

**Affiliations:** 1 Cardiology, Lisie Hospital, Kochi, IND; 2 Cardiology, North Eastern Indira Gandhi Regional Institute of Health And Medical Sciences, Shillong, IND; 3 Cardiology, Caritas Hospital, Kottayam, IND; 4 Cardiology, Madras Medical Mission Hospital, Chennai, IND; 5 Cardiology, Meitra Hospital, Edakkad, IND

**Keywords:** coronary bifurcation lesions, systematic two-stent techniques, provisional stenting technique, registry, nationwide

## Abstract

Background

Bifurcation coronary stenting (BCS) has unique therapeutic challenges. Several BCS strategies are prescribed for treatment, with conflicting data, and which is the best strategy for optimal short- and long-term outcomes remains a matter of debate. There is no systematic data from an Indian perspective in regard to patterns of BCS and its outcomes.

Methods and analysis

The Indian Bifurcation Stenting (IBIS) registry is a prospective, nationwide, endpoint-driven, investigator-initiated, multi-center, observational registry to compare the different bifurcation stent strategies, the effect of adjuvant techniques, and bifurcation anatomical differences in predicting short- and long-term clinical outcomes of bifurcation coronary interventions in India. A total of 1,000 patients from 20 clinical sites across the country will be enrolled in this study from September 2020 to August 2023. The primary endpoint will be the composite endpoint of major adverse cardiac events including cardiac death, target lesion myocardial infarction (MI), and ischemia-driven target lesion revascularization at the end of two years. The secondary endpoints include all causes of death, MI, target vessel revascularization, in-stent restenosis, stroke, and predefined procedural parameters. The safety endpoint is the occurrence of definite or probable stent thrombosis.

Conclusion

The aim of this prospective observational registry is to assess the practice patterns and clinical outcomes of patients undergoing coronary bifurcation lesion angioplasty in India. This will be extremely useful to provide an evidence-based insight as well as guidance to bifurcation angioplasty in India.

## Introduction

Approximately 15-20 % of all percutaneous coronary interventions (PCIs) involve bifurcation coronary stenosis [1,2], and it is often associated with technical challenges, lower procedural success, and worse clinical outcomes. There is no consensus regarding the appropriate strategy when treating coronary bifurcation stenosis (CBS). Multiple data from studies have provided an insight into different forms of stent implantation in CBS. The use of a single stent in provisional strategy to two stents in different techniques has been a challenge as to the choice of the strategy in different anatomical situations. A single-stent strategy is currently considered a standard stenting strategy for left main coronary artery bifurcation lesions because a two-stent strategy is associated with higher rates of adverse events such as target lesion revascularization (TLR) and stent thrombosis (ST) [3,4]. Additional techniques such as a final kissing balloon (FKB) and proximal optimization technique (POT) have resulted in better outcomes in bifurcation coronary stenting (BCS) [3,5]. Provisional stenting (PS) is the commonest and an approach by default in most of the bifurcation lesions, particularly when the side branch (SB) is small (diameter < 2.0 mm) and the main branch lesion length is small (usually <5 mm in length) [6-10]. But the effectiveness of PS for SBs more than 2.5 mm in diameter and the main vessel lesion length of more than 5 mm is underreported [11,12]. Moreover, currently, there are no angiographic criteria to differentiate the simple from complex CBS.

CBS vary in anatomy, plaque burden, the angle between branches, size of branches, and plaque shift post-angioplasty. Therefore, no two bifurcations are the same and a single strategy cannot be recommended for all bifurcations. There are many classifications for CBS and the most accepted one is the MEDINA classification. CBS is considered as true bifurcation stenosis when the SB is involved. A single-stent or PS strategy is planned in non-true bifurcations or when the SB is not relevant. Two-stent strategies are complex and indicated when the SB, which subtends a significant territory has high-grade stenosis. A number of studies have shown no additional benefit with a two-stent strategy over a single-stent or provisional strategy [13-16]. In addition, two-stent strategy is associated with more fluoroscopy, contrast volume, and biomarker release. However, there are CBS that warrant two-stent strategies from the outset depending on the lesion anatomy, plaque distribution, and bifurcation angle.

A provisional single-stent technique consists of stent implantation to the main vessel only with balloon angioplasty to the SB if the result is sub-optimal. However, in the case of suboptimal SB outcome, it may be necessary to stent the SB, converting the one-stent strategy to a two-stent technique. Various techniques for an upfront two-stent strategy have been described aiming at improving clinical outcomes. However, the best bifurcation strategy is still a matter of debate [17,18]. A recent meta-analysis found double kissing crush to be associated with fewer major adverse cardiac events (MACEs), compared to other two-stent techniques: Crush, Culotte, and T-Stenting/T and protrusion (TAP) [19].

Rationale of the study

BCS poses a therapeutic challenge and is associated with a worse clinical outcome and higher rates of peri-procedural and long-term complications. In India, approximately 0.7 million stents are implanted annually, but there is total lack of well-structured registry-based real-life data on CBS management. The National Interventional Council data of 2018 [20] showed that 4.32% of all coronary interventions in the country were BCS (total interventions: 438,351). This registry is planned to analyze the practice patterns regarding the treatment of BCS and outcomes of different techniques utilized for CBS in India. This outcome-based multi-center prospective observational registry, which is the first of its kind in our country, should provide proper information on the current prevailing BCS techniques and their impact on long-term clinical outcomes.

Objective of the study

The objective of the study is to compare the different bifurcation stent strategies, the effect of adjuvant techniques, and bifurcation anatomical differences in predicting short- and long-term clinical outcomes of bifurcation coronary interventions in India.

Primary endpoints

The composite endpoint of major adverse cardiac events (MACEs) includes cardiac death, target lesion myocardial infarction (MI), and ischemia-driven TLR at two years.

Secondary endpoints

Secondary clinical endpoints include in-hospital events as well as events at 1, 12, 24, and 36 months, which include cardiac death, all-cause mortality, and MI, target lesion MI (TLMI), TLR, ST, target vessel revascularization, any revascularization, and angina.

Secondary procedural endpoints at in-hospital and at 1, 12, 24, and 36 months include the number of stents implanted in BCS, total stent length in BCS, drug-eluting balloon, bifurcation strategy, SB rewiring, FKB, POT, POT-SB-POT (PSP) technique, and imaging modalities utilized for the procedure.

## Materials and methods

Study design and methods

**Figure 1:**
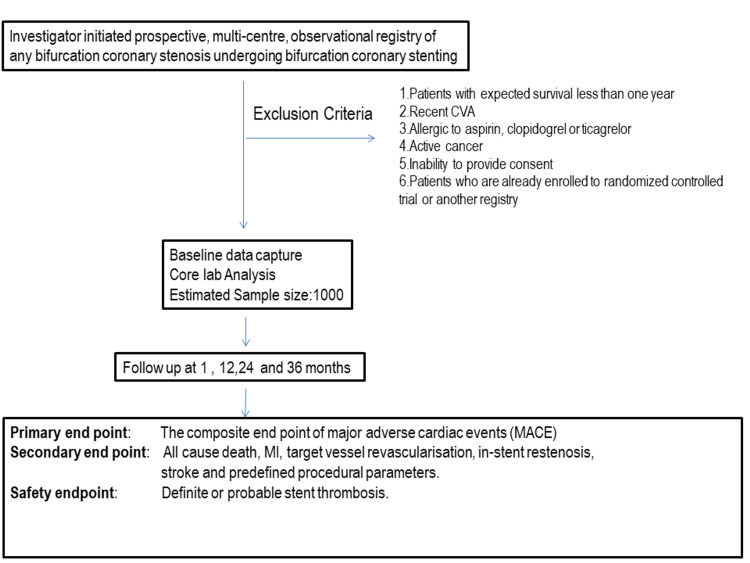
Overall study design and endpoints for the Indian Bifurcation Stenting Registry CVA, cerebrovascular accident; MI, myocardial infarction

This is an investigator-initiated prospective, multi-center, observational registry. Approximately 1,000 patients from 20 clinical sites will be enrolled in this study across India from September 2020 to August 2023. The selection of centers is based on a feasibility questionnaire send to the high-volume PCI centers across the country. All patients must meet all of the inclusion and exclusion criteria to be registered for the study (Figure 1).

The trial is prospectively registered with the Clinical Trials Registry - India (CTRI/2020/08/027411).

Inclusion criteria

The inclusion criteria include the following:

1. Age more than 18 years

2. Any bifurcation coronary stenosis undergoing BCS

3. Two or bifurcations in the same patients are eligible, as long as only one bifurcation per vessel

4. Patients with another single lesion in different vessels could be treated as indicated

Exclusion criteria

The exclusion criteria include the following:

1. Patients with an expected survival of less than one year

2. Recent cerebrovascular accident

3. Allergy to aspirin, clopidogrel, or ticagrelor

4. Active cancer

5. Inability to provide consent

6. Patients who are already enrolled in randomized controlled trials or another registry

Study methodology

All eligible patients with significant CBS in at least one native coronary artery after angiography will be prospectively enrolled in the study. The angiographic data of the CBS will be documented by Quantitative Coronary Analysis (QCA) and the intended bifurcation strategy will be noted.

Post-procedure, the bifurcation strategy (one stent or two stents) utilized will be documented with the stent data and need for adjuvant devices such as a scoring balloon, rotablation, or intra-vascular lithotripsy. In addition, the sequential steps for one-stent or various two-stent strategies for bifurcation stenting and optimization will be recorded and include rewiring, final kissing, and POT or PSP. The use of coronary imaging for optimization of bifurcation stenting will also be recorded. The type of strategy for CBS and usage of intracoronary imaging will be at the operator's discretion.

The participants will be made aware of the fact that they are free to discontinue the study at any point in time, ask any queries related to the study which pertains to them, and will be given enough time to consider the information provided. The signed and dated patient informed consent will be obtained before any specific procedure for the study is performed. The investigator will store the original, signed patient informed consent form. A copy of the signed patient informed consent form will be given to the patients.

Core lab analysis

The angiographic quantitative coronary analysis will be performed for all patients enrolled in the study

Data quality and management plan

Privacy of Personal Data

The study will collect and process only the data required for fulfilling the objectives of the study, and no other personal data will be collected. All data will be dealt with in compliance with the applicable data privacy protection laws and regulations of the country. The informed consent includes explicit consent to processing the personal data and allowing direct access to his/her original medical records (source data/documents) for study-related monitoring, audit, Independent Ethics Committee/Institutional Review Board review, and regulatory inspection.

Data Reporting

Data should be recorded from each site to an electronic case report form, which is accessed by each investigator with the unique ID & password provided to him. Etrewo®, which is a secure web-based application for building and managing online databases, will be used for data management. The database will be maintained by BioQuest Solutions Private Limited (Bengaluru, Karnataka, India). Access will be via a secure website from each participating site.

The principal investigator of each site should ensure the accuracy, completeness, and authenticity of the data collection. Electronic case report form entries are ensured with the maintenance of anonymity of the data. Central data management will be done by the national principal investigator at Lisie Hospital, Kochi, Kerala, India. The source documents will be checked by designated clinical research assistants to verify the correctness of data entered into the eCRF (electronic case report form).

## Results

Statistical analysis plan

The two-sample t-test or the Mann-Whitney U-test will be utilized to compare the continuous variables depending upon the distribution. The chi-square test or Fisher exact will be utilized to analyze the categorical variables depending on the cell numbers. Adjusted and unadjusted Cox regression based on the intention-to-treat principle will be utilized to estimate the hazard ratio, which is the main effect measure. The final follow-up date for the primary endpoints will be as follows: a median of three years of follow-up is achieved and the last enrolled patient has been followed for at least one year. All the defined endpoints will be reviewed until death or lost to follow-up, and both intention-to-treat and per-protocol analyses will be utilized to access the combined outcomes. Cox proportional hazard regression analysis will be utilized to read the effects of baseline differences between groups. A two-sided p-value of less than 0.05 indicates statistical significance.

## Discussion

Ethical considerations

This study will be conducted in compliance with the ethical principles that have their origin in the Declaration of Helsinki and its revisions, the International Conference on Harmonization guidelines for Good Clinical Practice, and all federal and local laws. The investigator will be responsible for ensuring that this study is conducted according to the investigational plan and for protecting the rights, safety, and welfare of study patients under the investigator's care. This is a prospective study of human research. No experimental drug, procedure, or intervention is involved in the protocol. However, if any ethical issues arise during the study, the Institutional Ethics Committee will be consulted and their recommendations followed.

Withdrawal of patients

Investigator Withdrawal of Patients

If the principal investigator of the site determines that the best medical interest of the patient is not to continue within the study, then he/she may withdraw a patient from the study following due procedure.

Patient Withdrawal From the Study

If a patient decides to withdraw from the study, it will not affect their treatment. The patient will be advised to discuss their concern in detail with the investigator and can decide to withdraw from the study if they are willing to do so. In the case where the patient is lost to follow-up, effort must be made to contact the patient and determine the possible reason for discontinuation/withdrawal and documented. Data from patients who have withdrawn from the study will be included in the final data analysis, up to the point of their withdrawal.

Adverse Event Reporting

All serious adverse events, whether suspected to be related to the device or not, must be reported within 24 hours of obtaining knowledge of the event.

## Conclusions

IBIS is the first nationwide registry intending to document the patterns of treatment of CBS and the outcome of BCS across India. This is the first planned registry that will provide organized data about CBS outcomes in our country where approximately 0.7 million stents are implanted annually but no systematic data exist about the patterns of treatment and outcomes.
